# Three-dimensional Reconstruction of Renal Vascular Tumor Anatomy to facilitate accurate preoperative planning of partial nephrectomy

**DOI:** 10.37796/2211-8039.1078

**Published:** 2020-12-01

**Authors:** Wei-Ching Lin, Chao-Hsiang Chang, Yi-Huei Chang, Chien-Heng Lin

**Affiliations:** a Department of Medical Imaging, China Medical University Hospital, Taichung, 40447, Taiwan; b College of Medicine, China Medical University, Taichung, 40402, Taiwan; c Department of Urology, China Medical University Hospital, Taichung, 40447, Taiwan; d Division of Pediatric Pulmonology, China Medical University Children's Hospital, 40447, Taiwan

**Keywords:** 3D reconstruction, Renal neoplasm, Partial nephrectomy

## Abstract

**Objectives:**

To evaluate the role of three-dimensional (3D) reconstruction tumors and vessels of the kidneys in aiding the preoperative planning of partial nephrectomy.

**Materials and methods:**

Patients with renal tumors to be treated with partial nephrectomy were included. Each patient underwent a preoperative computed tomography (CT) survey, and the reconstruction of each patient’s 3D arteriography and 3D surface-rendered tumor was performed based on the CT images for preoperative surgical planning.

**Results:**

A total of 6 patients, three with tumors of the right kidney and three with tumors of the left kidney, were enrolled in the study. The patients’ mean age was 49.33 ± 4.03 years (range: 45–57 years), and their mean tumor size was 4.4 ± 1.84 cm (range: 2.2–6.8 cm). Four underwent robot-assisted laparoscopic partial nephrectomies, one underwent a traditional laparoscopic partial nephrectomy, and one underwent a radical nephrectomy through laparotomy. Their average postoperative hospital stay was 6.7 days (range: 3–10 days). No intraoperative or postoperative complications were noted. The renal function was preserved in all the patients, and none of the patients exhibited evidence of local recurrence during more than 6 years of follow-up.

**Conclusions:**

3D arteriography fused with 3D surface-rendered tumor image navigation facilitates precise preoperative planning.

## 1. Introduction

Renal cell carcinomas (RCCs) are the most common type of renal tumors in adults, accounting for approximately 90–95% of all cases. The gold standard management is either partial or radical nephrectomy, depending on the anatomic location/size of the tumor, the tumor stage, or other case-specific factors [1–7]. Partial nephrectomy is recommended when feasible, and it is often performed to remove single tumors less than 4 cm in size and can sometimes be performed for tumors up to 7 cm in size. It can achieve oncologic outcomes than equivalent to those of radical nephrectomy, and in addition, it preserves renal function to a greater degree [8–10]. Nephron preservation is very important for patients with RCC since the five-year disease survival rate is about 80–95% in patients with stage I-II disease [7].

A detailed picture of the vascular tumor anatomy is critical in the preoperative planning for renal tumors, especially for cases in which a partial nephrectomy will be performed. Therefore, an image study is necessary and plays an important role [1,11,12]. Computed tomography (CT) and magnetic resonance imaging (MRI) have traditionally been used, and the images they generate generally consist of bi-dimensional cross-sections. However, three-dimensional (3D) reconstruction of cross-sectional images has been advancing for decades, and such 3D imaging provides more precise views of anatomical structures that facilitate accurate preoperative planning [2–4]. The thorough understanding of the vascular and tumor anatomy in a kidney is important to avoid injury to the renal parenchyma and major vessels and for achieving complete tumor excision.

The aim of this study was to evaluate the efficiency of using 3D reconstruction images of vascular tumor anatomy in the preoperative planning of possible partial nephrectomies for small renal tumors.

## 2. Material and methods

The hospital's institutional review board concurred with the view that this retrospective study was a continuous quality improvement initiative to improve patient care and that it thus did not require informed consent. This study was approved by the IRB of our hospital (**CMUH109-REC1-066**).

Patients with solitary renal tumors with tumor sizes <7 cm who were considered for partial nephrectomy, underwent a preoperative dynamic computed tomography (CT) survey, and were treated from September 2012 to December 2013 were included.

**Fig. 1:**
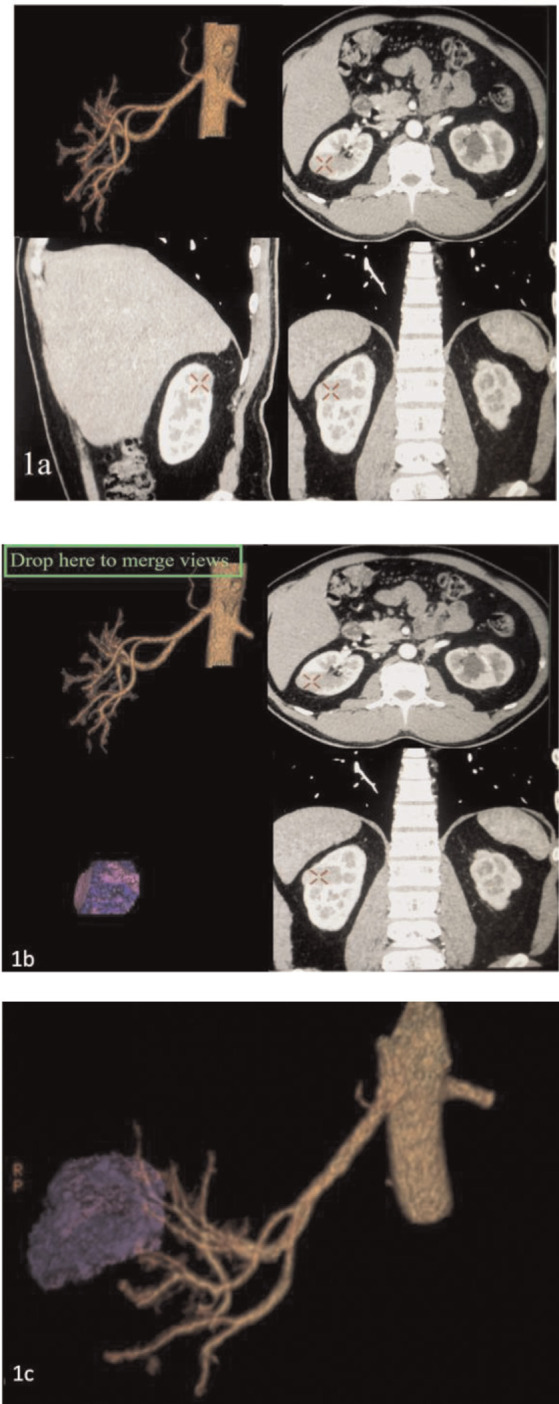
Schematic views of the process of creating a 3D reconstruction of renovascular tumor anatomy from CT images. (a) The 3D arteriogram (upper left) was created by using the growing method with a seed point on the main renal artery in early arterial phase axial, coronal, and sagittal CT images (upper right, lower right, and lower left). (b) The 3D surface-rendered tumor (lower left) was reconstructed using a cutting method that only preserved the tumor. (c) The 3D surface-rendered tumor image was combined with the 3D arteriogram and to create a combined display of the 3D arteriogram and the 3D surface-rendered tumor (purple nodule).

**Fig. 2:**
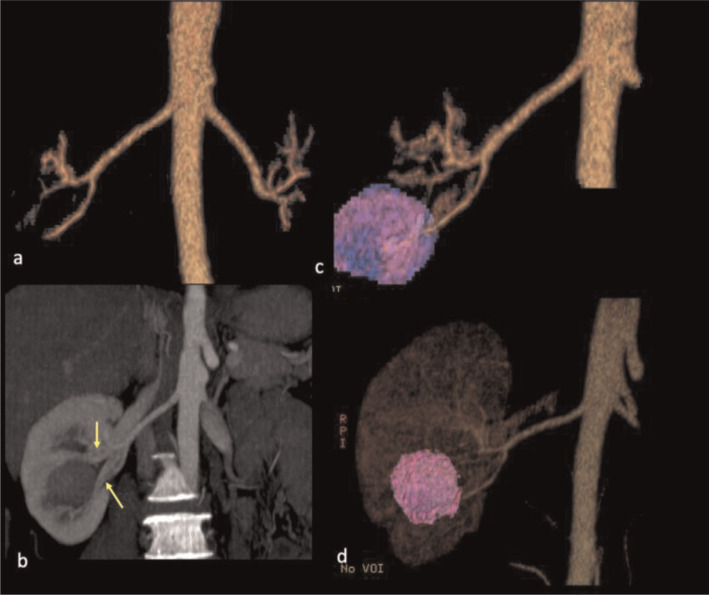
A 46-year-old female (case 4) had a 2.2 cm right renal tumor. (a) The 3D CT arteriography of the aorta and bilateral renal arteriogram was reconstructed first. (b) The maximum intensity projection (MIP) showed the relationship of the poorly enhanced renal tumor and adjacent renal venous branches (arrows). (c) The 3D CT angiography was merged with the 3D-surface rendered tumor to show that the blood supply of the renal tumor was from the inferior segmental artery. (d) The 3D CT angiography was merged with the 3D surface-rendered tumor and renal shadow to demonstrate the location of the tumor in the right kidney.

A dynamic contrast medium-enhanced CT scan was performed for each patient. More specifically, a 64-multidetector row CT scanner (LightSpeed VCT, GE Medical Systems, Milwaukee, WI) was used for four cases, while a 16-multidetector row CT scanner (BrightSpeed CT, GE Medical Systems, Milwaukee, WI) was used for two cases. For each patient, a total of 100 ml of non-ionic contrast medium was injected intravenously through a peripheral venous catheter at a rate of 3 ml/s. Scanned images of the arterial phase, corticomedullary phase, and nephrogenic phase were obtained at 15–25, 30–40, and 80–120 s, respectively. The raw slice thickness was 1.25 mm and reconstruction to 5 mm was conducted for the on axial and coronal sections. The 3D arteriography, surface-rendered 3D reconstruction of the tumor and kidney, and maximum intensity projection (MIP) for the renal vein were reconstructed (Fig. 1). The 3D arteriography and surface-rendered 3D reconstruction of the tumor and kidney were then combined to show the anatomic relationships of the tumor, arteries, and kidney (Fig. 2).

Continuous variables were expressed as means ± standard deviations, and because the amount of data was limited and not normally distributed, these variables were analyzed using the Wilcoxon test (paired samples). The SPSS package, version 11 (SPSS, Inc., Chicago, IL, USA) was used for analyses, and *p* values less than 0.05 were considered statistically significant to reject the null hypothesis.

## 3. Results

A total of 7 patients (five males and two females) were initially enrolled in the study. However, one of those patients was subsequently excluded due to brain metastasis and because a nephrectomy was not performed. Among the remaining 6 patients, three had tumors of the right kidney, and three had tumors of the left kidney. The patients’ mean age was 49.33 ± 4.03 years (range: 45–57 years, median: 48.5 years), and their mean tumor size was 4.4 ± 1.84 cm (range:2.2–6.8 cm, median: 4.4 cm). Three cases involved tumors with tumor sizes <4 cm, and the other three cases involved tumors with tumor sizes between 4 and 7 cm.

Four patients underwent robot-assisted laparoscopic partial nephrectomies, one underwent a traditional laparoscopic partial nephrectomy (case 5), and one underwent a traditional laparoscopic radical nephrectomy (case 6). The mean blood loss was 125 ± 47.87 ml (median: 150 ml). The average operation time for all the patients was 233.33 ± 31.97 min (median: 250 min). The warm and cold ischemia times were 25.5 and 18.5 min (median: 26.5 and 18.5 min), respectively, and the average total ischemia time was 33.33 ± 11.12 min (median: 27.5 min). The average postoperative hospital stay was 6.7 ± 1.1 days (range: 3–10 days). The average creatine levels before and after surgery were 0.91 ± 0.14 and 0.98 ± 0.17 mg/dl, respectively, while the median creatine levels before and after surgery were 0.935 mg/dl (95% CI: 0.69–1.07) and 0.92 mg/dl (95% CI: 0.71–1.17) respectively (*p*-value = 0.2188). The mean GFR values before and after surgery were 81.33 ± 8.84 and 77.5 ± 13.48 ml/min/1.73 m^2^, respectively, while the median GFR values before and after surgery were 88.5 ml/min/1.73 m^2^ (95% CI: 73.33–97.81) and 92.5 ml/min/1.73 m^2^ (95% CI: 66.52–96.42), respectively (*p*-value = 0.5625). The patients’ characteristics are shown in Table 1 and Table 2.

No intraoperative or postoperative complications, such as fistula, pseudoaneurysm, hematoma, urine leakage, or infection, were noted. The renal function was preserved in all six patients (Table 2). Furthermore, none of the patients exhibited evidence of recurrence during over 6 years of follow-up. One patient (case 2), however, had a newly grown RCC in the contralateral kidney 2 years after he received his first partial nephrectomy, and he then successfully underwent another partial nephrectomy to treat this tumor.

## 4. Discussion

Partial nephrectomy is preferred for the treatment of localized, small, early-stage renal tumors. It is not a good option, however, if the tumor is in the middle pole of the kidney, if the size of the tumor is very large, if there is more than one tumor in the same kidney, or if the cancer has spread to the lymph nodes or distant organs. Otherwise, a partial nephrectomy should only be performed by an experienced surgeon. Partial nephrectomy results in a lower risk of subsequent chronic kidney disease and thus enhances the survival rate in comparison to radical nephrectomy [13–16]. In this study, all of the patients except one (case 6) underwent partial nephrectomies. Initially, partial nephrectomy was preferred for this patient, but because the tumor was nearly 7 cm (6.8 cm) in size and was located deep in the middle pole of the left kidney, this was reconsidered, and after a review of the patient's 3D reconstruction images, a radical nephrectomy was performed for complete tumor resection and to avoid major vascular injury and urine leakage.

With the help of the 3D reconstruction technique, the small vessels around the lesion or in the tumor can be well visualized in the preoperative images, which can in turn help the surgeon preoperatively visualize the relationship between the tumor and adjacent arteries, prompt quick ligation of the feeding arteries, diminish the blood loss during the operation, avoid causing injury to the small vessels when removing the tumor or reconstructing the kidney, and shorten the warm ischemia time for hemostasis [17–20]. Thus, the postoperative hospital stay can be decreased, as can the likelihood of immediate operative complications or postoperative complications. In this study, in fact, there were no immediate operative complications or postoperative complications, and the warm ischemia time and post-operative hospital stay were 25.5 min and 6.7 days, respectively, which were similar to those reported in other studies [7,9,13].

The maximal preservation of renal function remains a priority in performing a partial nephrectomy, and such preservation is influenced by several factors, including case selection, preoperative renal function, surgical technique, and the volume of healthy renal parenchyma [21–23]. Wang et al. previously reported that a 3D reconstruction technique allowed for the accurate reconstruction of the relevant anatomical structures and presented the tiny vessels and tissues around a tumor, such that the technique proved to be a beneficial method in terms of preserving more parenchymal mass and postoperative ipsilateral GFR [17]. Furthermore, this technique can provide improvements that facilitate the performance of zero-ischemia robotic and laparoscopic partial nephrectomies, especially in patients requiring nephron sparing surgery [10], although the prolonged warm ischemia time was not significantly associated with long-term renal function after partial nephrectomy in a recent study [23–26]. Although we could not achieve zero ischemia time in this premilitary study, we would like to achieve zero ischemia time surgeries to achieve maximal renal function preservation in the future.

There was no local recurrence among the 6 patients in this study during more than 6 years of follow-up. One of the cases, case 2, was a patient who had a right renal tumor and underwent partial nephrectomy; however, he then had a left renal tumor 2 years later. The initial complete resection of the first tumor with a partial nephrectomy preserved the patient's renal function; thus, he could also undergo the second partial nephrectomy.

In the rendering technique used in this study, axial CT image data was serially transformed into 3D images. Volume rendering always accurately depicts 3D relationships, while MIP, a specific type of rendering in which the brightest voxel is projected into the 3D image, has become a valuable tool for 3D rendering of the vasculature [27–31]. MIP may allow the visualization of smaller branch vessels with less work than is required for volume rendering [32,33]. Combining volume-rendered and MIP images enables a comprehensive understanding of the full extent of the relationships among the tumor, renal arteries, renal veins, and kidney [34].

More recently, 3D-printed models have been use in surgical planning for laparoscopic partial nephrectomies [8,35,36]. Such models have the lifelike transparency and colors of the kidney, tumor, main arteries, and vessels, and with each element shown in a different color, provide accurate pictures of what surgeons will see during operations in all three dimensions, greatly facilitating surgical planning and allowing damage to delicate arteries and vessels and the risk of a patient's kidney needing to be completely removed to be avoided [37]. These advantages are not easily achievable with a 2D scan. The method we used in this study was similar to that of using 3D-printed models, except that the reconstruction images were not printed. Both approaches require relatively long amounts of time for post processing; however, this is especially true for the 3D printing process, as it can take up to 30 h to print a 3D kidney model, and even as much a few days for the entire 3D printing process to be completed [37,38]. Besides, 3D-printed models require extra equipment and extra costs to print the models [39]. That said, 3D-printed models showing the anatomies of the kidney and tumor can make it easier for surgeons to understand the oncologic conditions of patients, decreasing the need for surgical experience [40].

### 4.1. Limitations

There were some limitations in this study. First, it was a retrospective, single-center study, the number of cases was relatively few, and we analyzed this small number of cases without comparing them to cases in which 3D reconstruction CT images or 3D-printed models were not used. However, the study did include very long-term follow-up data that allowed us to better see the effects of the surgeries since patients with stage I RCCs have a five-year survival rate of 80–95%. Nonetheless, a study with a larger population with different comparison models and long-term follow-up should be conducted in order to further assess the relevant outcomes.

## 5. Conclusion

The technique of using 3D reconstructed images combined with 3D arteriography and 3D surface-rendered tumors used in this study can facilitate maximal postoperative renal function preservation after partial nephrectomy, especially in patients requiring nephron sparing surgery. In addition, 3D printing should be further developed in the future to decrease the rate of errors and complications during the actual surgery and possibly improve morbidity and mortality.

## Figures and Tables

**Table 1:** Patient characteristics.

Case	Age/Sex	Diagnosis	Location	Size (cm)	Ischemia time (warm + cold) (min)	Blood loss (ml)	Operation time (min)
1	51/M	Renal clear cell carcinoma, grade 2	Left	5.8	28	200	250
^2^	47/M	Renal clear cell carcinoma, grade 2	Right	6	33 (22 + 11)	100	270
3	50 M	Papillary renal cell carcinoma, grade 3	Right	2.6	21	100	250
4	46/F	Papillary renal cell carcinoma, grade 2	Right	2.2	53 (27 + 26)	50	180
5	57/M	Renal clear cell carcinoma, grade 2	Left	3	30	150	250
6	45/F	Renal clear cell carcinoma, grade 1	Left	6.8	25	150	200

M = male, F = female.

**Table 2:** Patient characteristics.

Case	Creatine (mg/dl)	eGFR (mL/min/1.73 m^2^)	Creatine (mg/dl) after operation	eGFR (mL/min/1.73 m^2^) after operation
1	1.1	71	1.2	64
^2^	0.97	83	0.93	97
3	0.95	84	1.02	77
4	0.65	98	0.67	94
5	0.97	79	1.17	64
6	0.84	73	0.88	69

eGFR = estimated glomerular filtration rate.
